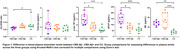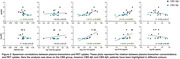# Relationships between PET and blood plasma biomarkers in corticobasal syndrome

**DOI:** 10.1002/alz.094001

**Published:** 2025-01-09

**Authors:** Neha Atulkumar Singh, Alla Alnobani, Jonathan Graff‐Radford, Mary M. Machulda, Michelle M. Mielke, Christopher G. Schwarz, Matthew L. Senjem, Clifford R. Jack, Val J. Lowe, Takahisa Kanekiyo, Keith A. Josephs, Jennifer L. Whitwell

**Affiliations:** ^1^ Mayo Clinic, Rochester, MN USA; ^2^ Mayo Clinic, Jacksonville, FL USA; ^3^ Wake Forest University School of Medicine, Winston‐Salem, NC USA

## Abstract

**Background:**

Corticobasal syndrome (CBS) is a progressive neurodegenerative syndrome that can result from several underlying neuropathologies, including Alzheimer’s disease (AD). Little is known about the utility of blood plasma measures to predict PET biomarker confirmed AD in CBS.

**Method:**

Eighteen CBS patients (8 PET Aß+; 10 PET Aß‐) and 8 cognitively unimpaired Aß‐ individuals (CU) were recruited by the Neurodegenerative Research group from the Department of Neurology, Mayo Clinic, Rochester, MN, underwent Aß (Pittsburgh Compound‐B) and tau (18F‐flortaucipir) PET and provided a blood sample. Blood plasma analysis was performed to assess p‐tau 181, total tau, Aß 42/40, neurofilament light chain (NfL) and glial fibrillary acidic protein (GFAP) using the Quanterix Simoa platform. Plasma concentrations were compared between Aß‐, and Aß+ CBS and controls using Kruskal‐Wallis corrected for multiple comparisons using Dunn’s test, with effect size measured with area under the receiver operating characteristic curve (AUROC) analysis. Spearman correlations were calculated to assess relationships between plasma concentrations and Aß and tau PET uptake.

**Result:**

The CBS Aß+ group showed reduced Aß42/40 ratio, with elevated p‐tau 181, GFAP and NfL plasma concentrations compared to CU, while the CBS Aß‐ group only showed elevated NfL concentration compared to CU (Figure 1). Both p‐tau 181 (AUROC=0.95) and GFAP (AUROC=0.91) plasma concentrations were able to differentiate CBS Aß‐ from CBS Aß+. Plasma p‐tau 181 and GFAP concentrations showed a positive correlation to both Aß and tau PET uptake, and the Aß42/40 ratio showed a negative correlation to Aß PET uptake (Figure 2). No correlations were observed between NfL and Aß and tau PET.

**Conclusion:**

This study supports the use of plasma p‐tau 181 and GFAP to detect AD in CBS, while NfL shows potential as a nonspecific disease biomarker of CBS regardless of underlying pathology. Plasma concentrations of these biomarkers may potentially be incorporated into clinical trials targeting CBS patients with underlying AD pathology.